# Trabecular bone deterioration in a postmenopausal female suffering multiple spontaneous vertebral fractures due to a delayed denosumab injection – A post-treatment re-initiation bone biopsy-based case study

**DOI:** 10.1016/j.bonr.2023.101703

**Published:** 2023-07-22

**Authors:** Louise Alstrup Drejer, Bilal Mohamad El-Masri, Charlotte Ejersted, Christina Møller Andreasen, Lisbeth Koch Thomsen, Jesper Skovhus Thomsen, Thomas Levin Andersen, Stinus Hansen

**Affiliations:** a Department of Endocrinology, University Hospital of Southern Denmark, Esbjerg, Denmark; b Department of Pathology, Odense University Hospital, Odense, Denmark; c Department of Clinical Research, University of Southern Denmark, Odense, Denmark; d Department of Molecular Medicine, University of Southern Denmark, Odense, Denmark; e Department of Endocrinology, Odense University Hospital, Odense, Denmark; f Department of Biomedicine, Aarhus University, Aarhus, Denmark; g Department of Forensic Medicine, Aarhus University, Aarhus, Denmark

**Keywords:** Osteoporosis, Denosumab, Bone histomorphometry, μCT, Bone remodeling, Rebound, Bone resorption

## Abstract

**Background:**

Denosumab, is a potent anti-resorptive that, increases bone mineral density, and reduces fracture risk in osteoporotic patients. However, several case studies have reported multiple vertebral fractures in patients discontinuing denosumab.

**Case presentation:**

This case report describes a 64-year-old female with postmenopausal osteoporosis treated with denosumab, who had her 11th injection delayed by 4 months. The patient suffered eight spontaneous vertebral fractures. After consent, an iliac crest bone biopsy was obtained following re-initiation of the denosumab treatment and analyzed by micro-computed tomography and histomorphometry.

**Results:**

micro-computed tomography analysis revealed a low trabecular bone volume of 10 %, a low trabecular thickness of 97 μm, a low trabecular spacing of 546 μm, a high trabecular number of 1.8/mm, and a high structure model index of 2.2, suggesting trabecular thinning and loss of trabecular plates. Histomorphometric trabecular bone analysis revealed an eroded perimeter per bone perimeter of 33 % and an osteoid perimeter per bone perimeter of 62 %. Importantly, 88 % of the osteoid perimeter was immediately above an eroded-scalloped cement line with no sign of mineralization, and often with no clear bone-forming osteoblasts on the surface. Moreover, only 5 % of the bone perimeter was mineralizing, reflecting that only 8 % of the osteoid perimeter underwent mineralization, resulting in a mineralization lag time of 545 days. Taken together, this indicates limited bone formation and delayed mineralization.

**Conclusion:**

We present a case report of multiple vertebral fractures after denosumab discontinuation with histomorphometric evidence that denosumab discontinuation leads to extensive trabecular bone resorption followed by a limited bone formation and delayed mineralization if the denosumab treatment is reinitiated. This highlights the importance of developing optimal discontinuation strategies for patients that are to discontinue treatment.

## Highlights

• Multiple vertebral fractures following denosumab discontinuation are most likely due to extensive bone resorption.
• The resorbed bone surfaces following denosumab discontinuation are only partly replenished if treatment is reinitiated
• Newly formed osteoid exhibits delayed mineralization if denosumab treatment is reinitiated.

## 1 Introduction

Denosumab is a human monoclonal antibody that blocks the receptor activator of the nuclear factor kappa B ligand (RANKL) signaling pathway thereby inhibiting osteoclast differentiation, activity, and survival. In postmenopausal women, denosumab treatment lowers bone resorption, increases bone mineral density (BMD) at the spine and hip, and reduces vertebral and non-vertebral fracture occurrence (Cummings et al., 2009). Often BMD increases to levels above the osteoporotic range and in some patients, treatment is therefore discontinued. However, since 2015, several case reports have reported the occurrence of vertebral fractures after denosumab discontinuation (Anastasilakis and Makras, 2016; Aubry-Rozier et al., 2016; Polyzos and Terpos, 2016; Popp et al., 2016; Lamy et al., 2017; Anastasilakis et al., 2017). Subsequently, the consequence of discontinuing denosumab has been evaluated in post hoc analyses, using data from the randomized, placebo-controlled trial that documented denosumab's efficacy (FREEDOM study) and its extension (FREEDOM Extension) (Cummings et al., 2009; Bone et al., 2017; Cummings et al., 2018; McClung et al., 2017). It was found that after discontinuation, biochemical indices of bone resorption increased and surpassed pre-treatment values within months and then gradually declined. Moreover, the increases in BMD obtained with treatment vanished and the BMD returned to baseline values within two years off-treatment (Cummings et al., 2018; McClung et al., 2017; Bone et al., 2011; Miller et al., 2008). In addition, denosumab discontinuation was associated with an increased risk of vertebral fractures (Cummings et al., 2018; Lyu et al., 2020). Since then, attention has focused on the clinical management of bone loss and fracture risk after denosumab discontinuation and the optimal use of anti-resorptive therapeutics for this purpose (Tsourdi, 2021). However, only a few studies have addressed the effects on bone remodeling at the histological level and on bone microarchitecture in cortical and trabecular bone in those suffering from vertebral fractures after denosumab discontinuation. Such investigations are important for clarifying the pathophysiological mechanisms and could potentially guide the choice of subsequent anti-osteoporotic therapy when denosumab is discontinued.

This case report presents an extreme clinical case in which a younger postmenopausal female suffered eight spontaneous vertebral fractures after a delayed 11th denosumab injection. A transiliac bone biopsy was obtained 4 months after re-initiation of the denosumab treatment and analyzed using micro-computed tomography (μCT) and histomorphometry.

## 2 Materials and methods

### 2.1 Patient, clinical imaging, and biochemical characteristics

**Fig. 1:**
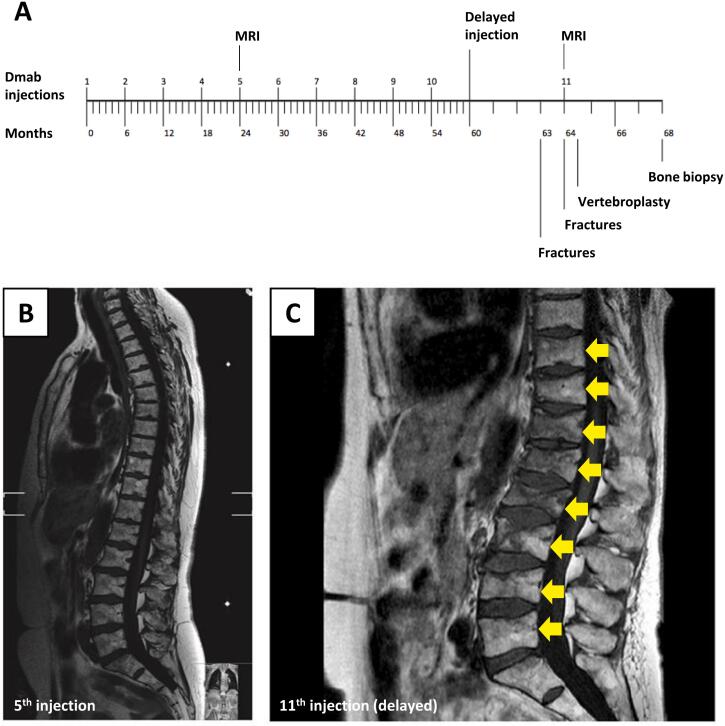
A 64-year-old Caucasian female with osteoporosis experiencing vertebral compression fractures following a delay of the 11th denosumab treatment. A: Timeline showing the patient's regular denosumab injections every 6 months and the 4 months delay of the 11th injection, causing multiple vertebral compression fractures at months 63 and 64. A transiliac bone biopsy was collected at month 68. B-C: Magnetic resonance images (MRI) were obtained at the 5th Denosumab injection (month 48) (B) and month 64, showing vertebral compression fractures (arrows) (C).

The patient was a 64-year-old Caucasian female who was diagnosed with postmenopausal osteoporosis seven years earlier i.e., at the age of 57 years. The medical history with results from X-ray and magnetic resonance imaging (MRI) of the spine, dual-energy X-ray absorptiometry (DEXA), and blood samples are presented. At the time of diagnosis, a DEXA scan showed a lumbar spine (L1-L4) T-score of −3.8 and a total hip T-score of −1.6. No vertebral fractures were found on a DEXA lateral spine radiograph. Previously the patient was identified as a human leucocyte antigen B27 (HLAB27) genotype and had been diagnosed with ankylosing spondylitis and treated with Eternacept. This led to remission and Eternacept was discontinued two years before the osteoporosis diagnosis. The patient had no family history of osteoporosis and had never had peripheral fractures apart from a fracture of a toe. The patient smoked cigarettes and had mild chronic obstructive pulmonary disease treated with inhaled steroids. An oral bisphosphonate (Alendronate 70 mg weekly) was prescribed but due to gastrointestinal side effects treatment was changed to denosumab 60 mg subcutaneously every six months (Fig. 1). A total of 10 denosumab injections were given and substantial increases in BMD were found on DEXA re-assessments after three years (lumbar spine/total hip T-scores:-3.2/-1.2) with similar levels after five years of treatment (lumbar spine/total hip T-scores: -3.2/-1.2).

By mistake, the 11th denosumab injection was delayed. Ten months after the 10th Denosumab injection, i.e. 4 months after the 11th denosumab should have been given, the patient was referred to the emergency department due to acute lower back pain. A spine x-ray revealed acute compression fractures of the first, third, and fourth lumbar vertebras. The patient was discharged with analgesics. One month later the patient was re-admitted with worsening back pain and a spine MRI revealed an additional five acute vertebral fractures of the vertebras Th10, Th11, Th12, L2, and L5. Strong analgesics were prescribed yet severe and debilitating pain persisted and the patient was referred to a surgical center. Percutaneous vertebroplasty with cement injection into the vertebral bodies at all eight levels was performed in two surgical séances and the patient experienced moderate pain relief in the following weeks. The patient suffered a 9 cm height loss.

Afterward, the 11th denosumab injection was given, and extensive evaluation for secondary causes of osteoporosis or malignancy was performed without positive findings. Similar to findings at the time of diagnosis biochemical assessments showed normal levels of calcium, phosphate, creatinine, parathyroid hormone, thyroid-stimulating hormone, and a 25-hydroxy-vitamin D of 61 nmol/l. The biochemical marker of bone resorption C-terminal cross-linking telopeptide of type 1 collagen (CTX) was 80 ng/l and the bone formation marker procollagen type 1 N propeptide (P1NP) was 68 μg/l (median and 95 % reference interval in pre-menopausal younger Danish women: CTX 264 (71–976 ng/l), and P1NP 41 (18–94 μg/l)) (Jorgensen et al., 2017).

Four months after the 11th denosumab injection and after consent an iliac crest bone biopsy was obtained and the patient started treatment with Teriparatide 20 mg s.c. daily that has been given for 20 months without the occurrence of further fractures.

### 2.2 Bone biopsy

The patient was given tetracycline hydrochloride 250 mg orally three times daily, 5–7 days and 17–19 days before a transiliac crest bone biopsy was obtained using a Bordier-Meunier trephine with a 7.5 mm diameter. The bone biopsy was fixed and stored in 70 % ethanol at 4 °C until dehydrated and embedded non-decalcified in methyl-methacrylate (Recker et al., 2018).

### 2.3 Micro-computed tomography (μCT)

The embedded bone biopsy was μCT-scanned (μCT35, Scanco Medical AG, Brüttisellen, Switzerland) with an isotropic voxel size of 6 μm, an Xray voltage of 55 kVp and current of 145 μA, and an integration time of 800 ms. After image reconstruction, Volumes of Interest (VOIs) were drawn in the trabecular compartment using custom software, as previously described in detail (Sikjaer et al., 2012). Standard microstructural parameters were determined using the software supplied with the scanner (Image Processing Language v5.15, Scanco Medical AG) and included bone volume fraction (BV/TV); trabecular number (Tb.N), thickness (Tb.Th), and separation (Tb.Sp); structure model index (SMI); connectivity density; and tissue mineral density. The microstructural parameters were compared with those of a reference population of Danish women for trabecular bone (*N* = 41, aged 19–96 years) (Thomsen et al., 2015).

### 2.4 Bone histomorphometry

Following μCT-scanning, the bone biopsy was serially sectioned into 7-μm-thick sections at several levels of the bone biopsy. A single section was stained with hematoxylin and eosin (HE), to visualize cells, while a Tartrate-resistant acid phosphatase (TRAcP) activity staining was carried out on a single section and on a positive control section to assess the presence of osteoclasts on the bone surface. TRAcP activity staining was carried out as described previously (van de Wijngaert and Burger, 1986). Static bone histomorphometry was performed within the trabecular, endocortical, and intracortical compartments, while dynamic histomorphometric analysis, was performed on the trabecular and endocortical compartments of the transiliac bone biopsy. The histomorphometric analysis was performed on three different sections each taken at different levels. Static histomorphometric analysis was performed on Goldner's trichrome stained sections scanned using polarized light, while dynamic histomorphometric analysis was performed on an adjacent unstained section that was scanned using fluorescence (tetracycline labels). The scans were performed using a fluorescence slide scanner (VS200, Olympus, Tokyo, Japan).

Trabecular and endocortical static 2-dimensional bone perimeters were determined, including the eroded perimeter/bone perimeter (E.Pm/B.Pm) reflecting the extent of bone resorption, and the osteoid perimeter/bone perimeter (O.Pm/B.Pm) reflecting the extent of bone formation. The O.Pm/B.Pm was further subdivided into two types i) O.Pm/B.Pm immediately above an eroded-scalloped cement line with no newly mineralized lamella bone present below (O.Pm/B.Pm^no-min^) and ii) O.Pm/B.Pm on top of newly mineralized lamella bone (O.Pm/B.Pm^min^). Additionally, trabecular and endocortical mineralizing perimeter/bone perimeter (M.Pm/B.Pm), mineral apposition rate (MAR), mineralization lag time (MLT), and osteoid maturation time (OMT) were determined using unstained sections as described in (Dempster et al., 2013).

The intracortical bone remodeling was assessed by classifying intracortical pores, according to their remodeling type and stage, as previously described (Andreasen et al., 2018a). Firstly, the pores were divided into two different remodeling types: Type 1 remodeling reflects the generation of a new canal, where its resorption area does not overlap with the pore of an existing osteon. Type 2 remodeling reflects the remodeling of an existing canal, where its resorption area overlaps with one or more pores of existing osteons. Secondly, the pores were classified according to their remodeling stage: i) Quiescent pores, exhibiting no ongoing bone remodeling (QS). ii) Eroded pores exhibiting eroded surfaces (ES), but without bone formation (OS). iii) Eroded-formative (EF) pores exhibiting both eroded surfaces and formative osteoid surfaces. iv) Formative pores, exhibiting osteoid surfaces and without eroded surfaces. Eroded, eroded-formative, and formative pores are collectively termed non-quiescent pores.

The pore diameter and area were measured for all pores, while the osteon diameter was measured for all quiescent pores and used to determine the wall thickness, as previously reported in (Andreasen et al., 2018b). The cortical porosity and area were determined by point counting, and the average cortical width was determined as cortical area divided by cortical length, as described in (Andreasen et al., 2018b). The relative contribution of the different pore types to the total pore area was ascertained. The intracortical parameters were compared to two reference populations: a cohort of 35 healthy women (aged 16–78 years) (Andreasen et al., 2018a; Bakalova et al., 2018) and a cohort of 17 women diagnosed with postmenopausal osteoporosis (PMO) (aged 58–80 years) (Andersen et al., 2013; Thomsen et al., 2021).

## 3 Results

### 3.1 μCT analysis reveals a deteriorated trabecular microarchitecture with thin rod-like trabeculae

**Fig. 2:**
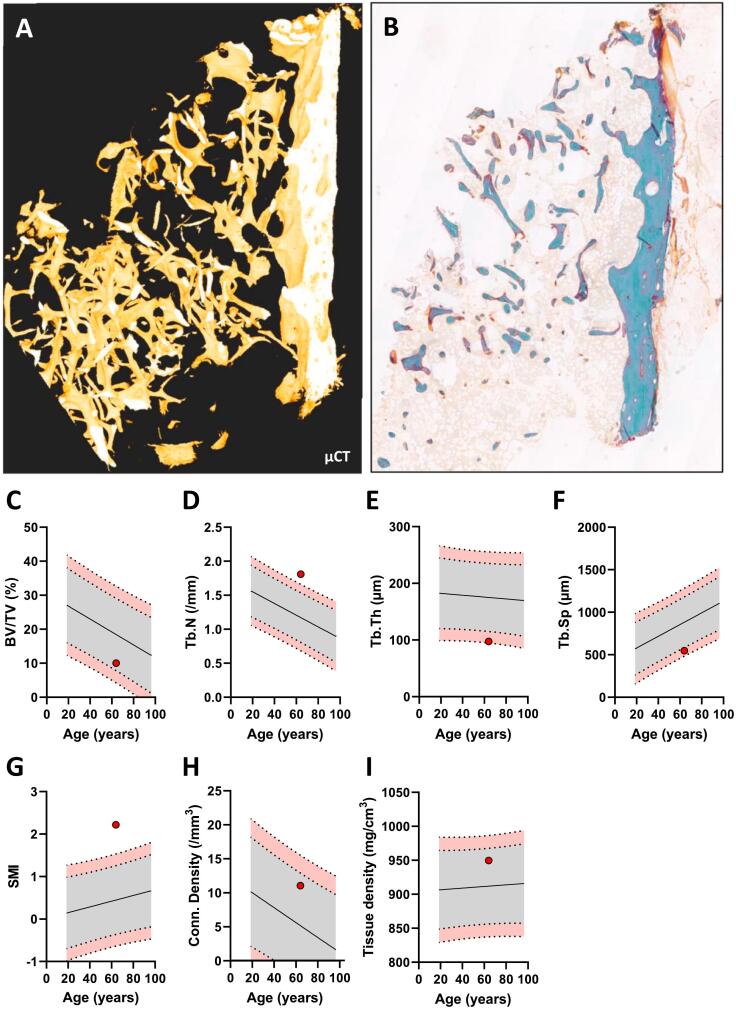
Microstructure of the transiliac bone biopsy. A-B: Micro-computed tomography (μCT) (A) and Goldner's Trichrome stained section (B) showing the microstructure of the bone biopsy. C-I: Microstructural parameters relative to a reference population of 41 women (REF): Trabecular bone volume (BV/TV) (C), Trabecular number (Tb.N) (D), Trabecular thickness (Tb.Th) (E), Trabecular spacing (Tb.Sp) (F), Structure model index (SMI) (G), Connectivity Density (H) and Tissue density (I). The curve represents a reference population of female controls (*n* = 41), and the dotted lines represent its 95 % and 99 % prediction bands.

The trabecular microstructural parameters were compared with those of a reference population of Danish women (*n* = 41, aged 19–96 years) (Thomsen et al., 2015). The patient presented with a shattered trabecular microstructure (Fig. 2A-B) and a low trabecular bone volume fraction of 10 % (Fig. 2C). Although this is within the 95 % prediction interval of the reference, the trabecular bone volume fraction nearly exceeds the lower 95 % reference limit, indicating a low trabecular bone volume fraction. Moreover, the patient had a high trabecular number (Tb.N) of 1.8/mm, a low trabecular thickness (Tb.Th) of 97 μm, a low trabecular spacing (Tb.Sp) of 547 μm and a high structure model index (SMI) of 2.2. (Fig. 2D-G). Taken together, this indicates a trabecular network mainly consisting of thin, rod-like trabeculae. Finally, the microstructural analysis revealed a connectivity density of 11 mm^−3^ and a tissue mineral density of 950 mg/cm^3^, although these did not differ notably from the reference as they were placed inside the 95 % prediction intervals of the reference population (Fig. 2H-I).

### 3.2 Osteoid surfaces lack a clear presence of bone-forming osteoblasts following denosumab treatment re-initiation

**Fig. 3:**
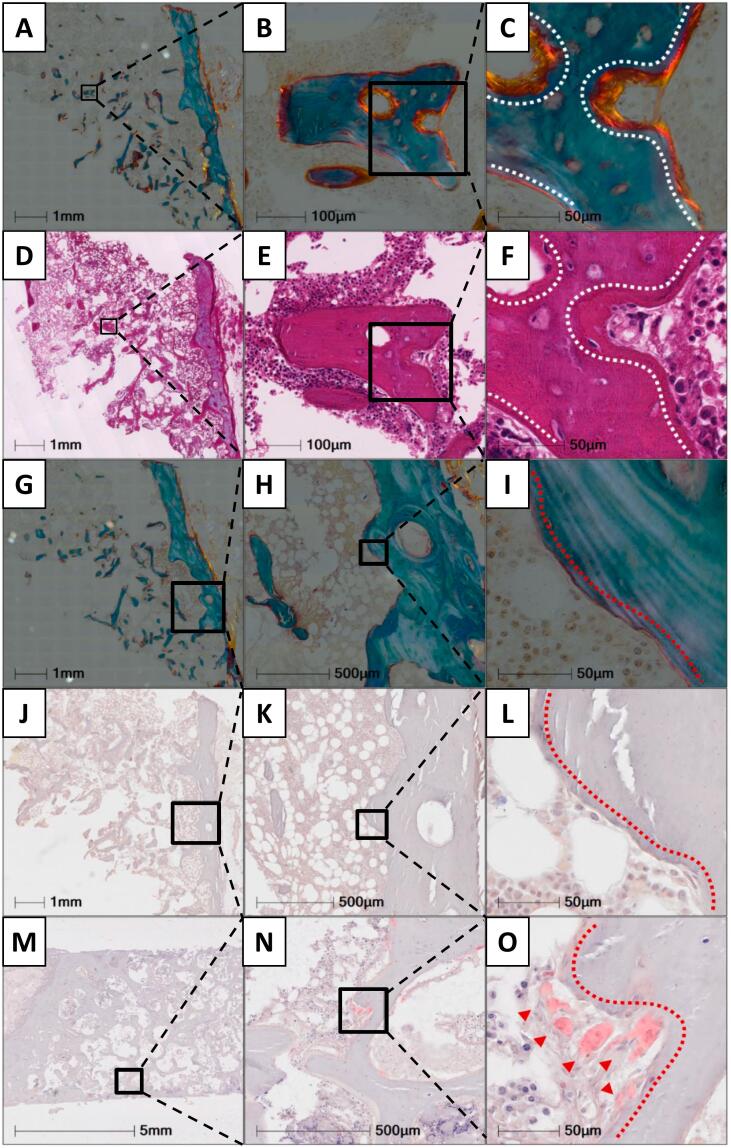
Goldner's trichrome (A-C & G-I), HE (E-H), and TRAcP activity stained bone sections. Goldner's trichrome-stained sections illustrate the trabecular osteoid perimeters (A-C) with an adjacent HE-stained section illustrating the absence of osteoblasts with a clear cuboidal morphology above osteoid surfaces (D-F). Moreover, eroded surfaces are illustrated on a Goldner's trichome stained section, with a broken lamella structure (G-E), while a TRAcP activity stained adjacent section illustrates the absence of osteoclasts (J-L). Finally, a positive TRAcP activity stain was included, where osteoclasts are visualized. White dashed lines highlight osteoid surfaces, red dashed lines highlight eroded surfaces and red arrows highlight osteoclasts.

A representative Goldner's Trichome stained section illustrates the presence of osteoid (Fig. 3A-C), while no clear cuboidal bone-forming cells located on the osteoid surfaces were detected on an adjacent HE-stained section (Fig. 3D-F). Thus, the lack of clearly appearing bone-forming osteoblasts on the bone formative surfaces indicates that bone formation was initiated, but not continued. Additionally, the TRAcP activity staining confirm the absence of osteoclasts on the eroded bone surfaces (Fig. 3G-O), in agreement with the CTX level detected to be below the median of pre-menopausal women.

### 3.3 The trabecular compartment exhibits an extended eroded and superficial osteoid perimeter with signs of a delayed mineralization

**Fig. 4:**
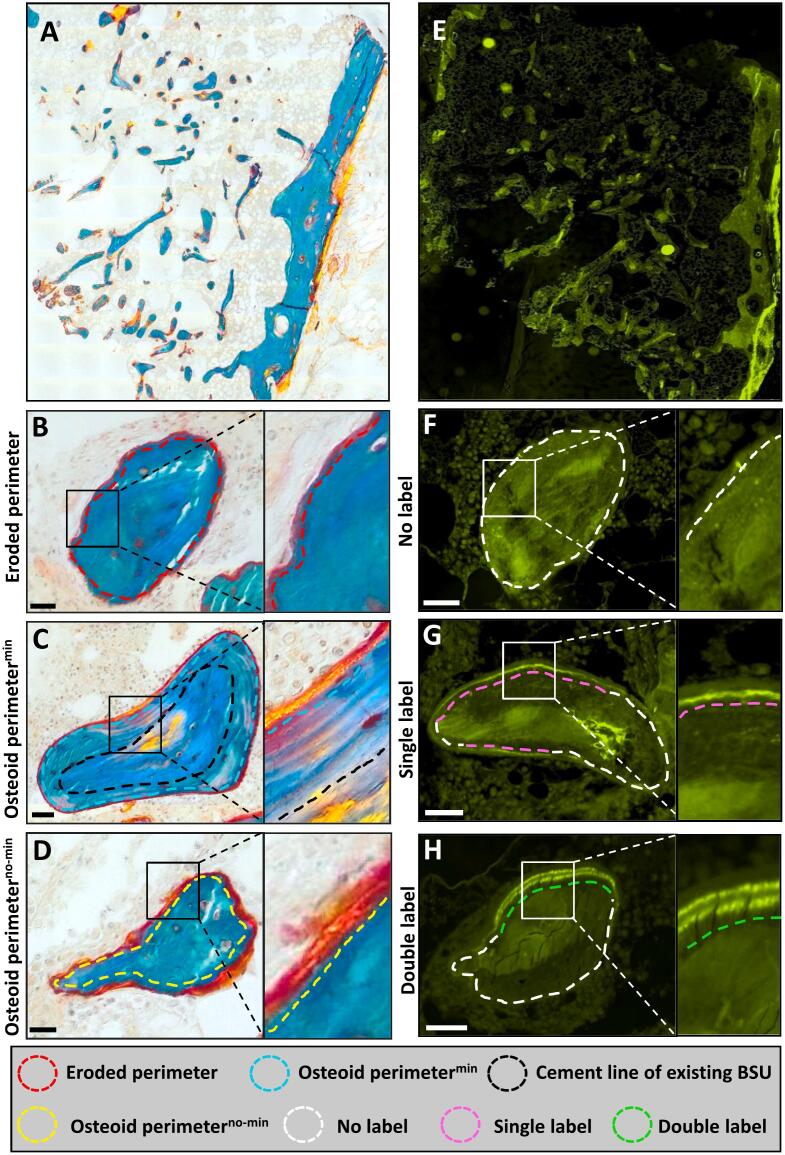
Goldner's trichrome stained (A-D), and Tetracycline labeled sections (E-H). The Goldner's trichrome stain illustrates the trabecular perimeters that are either eroded (B), Osteoid perimeters on top of newly mineralized lamella bone (Osteoid perimeter^min^) (C), or osteoid perimeters immediately above an eroded-scalloped cement line with no newly mineralized lamella bone present below (Osteoid perimeter^no-min^) (D). The tetracycline labeled section illustrates the trabecular perimeters with either no tetracycline label (F), with a single label (G), or with a double label (H).

**Fig. 5:**
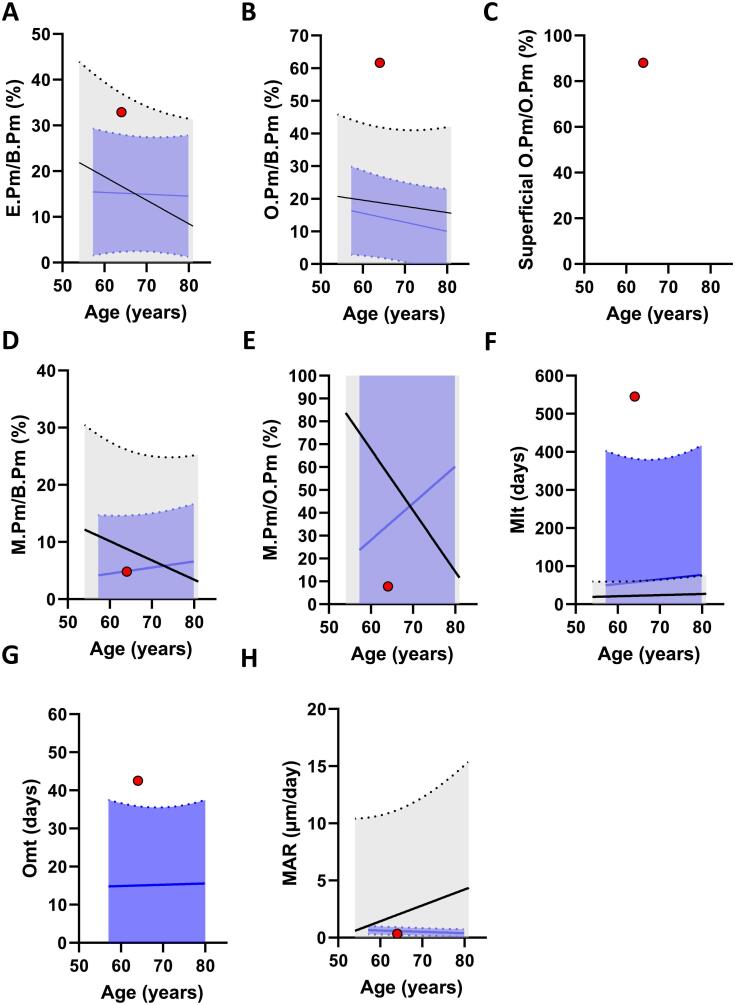
Trabecular bone histomorphometry. Goldner's trichrome stained sections were assessed for both Static (A-C) and dynamic (D-G) histomorphometry, relative to a reference population of female controls and postmenopausal women with osteoporosis, respectively: Eroded perimeter/bone perimeter (E.Pm/B.Pm) (A), osteoid perimeter/bone perimeter, (O.Pm/B.Pm) (B), mineralizing perimeter/bone perimeter (M.Pm/B.Pm) (D), mineralizing perimeter/osteoid perimeter (M.Pm/O.Pm) (E), mineral apposition rate (MAR) (F), mineralization lag time (Mlt). The black curve represents a reference population of female controls (*n* = 35) and the blue curve represents a reference population of postmenopausal women with osteoporosis (*n* = 17), and their dotted lines represent its 95 % prediction bands.

The histomorphometric parameters were compared to a reference population of healthy women and a reference population of post-menopausal women. Histomorphometric analysis revealed an extended eroded perimeter of 32.9 % (Fig. 4B, 5A) and an osteoid perimeter of 61.6 % (Fig. 4C, D, 5B). Of note, 88 % of the osteoid perimeter was superficial osteoid located directly on the eroded perimeter (Fig. 4D & 5C). This indicates that bone formation was initiated, but not continued (Fig. 5A-C). The mineralizing perimeter (M.Pm/B.Pm) was 4.8 %, while only 7.8 % of the osteoid perimeter was undergoing mineralization (M.Pm/O.Pm), resulting in a high mineralization lag time of 545 days, an osteoid maturation time of 43 days, and a mineral apposition rate of 0.34 μm/day (Fig. 4E-H, & 5D-H).

### 3.4 The endocortical compartment exhibits extensively eroded surfaces indicating cortical trabecularization

Histomorphometric analysis of the endocortical compartment revealed an eroded perimeter (E.Pm/B.Pm) of 67.1 % and an osteoid perimeter (O.Pm/B.Pm) of 22.8 % (Table 1). Moreover, 61.8 % of the endocortical osteoid perimeter was reflecting superficial osteoid directly on eroded surfaces (Table 1). Analysis of mineralization labels revealed that only 4.7 % of the bone perimeter was undergoing mineralization (M.Pm/B.Pm), meaning only 20.6 % of the osteoid perimeter was undergoing mineralization (M.Pm/O.Pm) (Table 1). This is also in line with the lack of cuboidal bone-forming osteoblasts on the osteoid surfaces (Fig. 3D-F).

**Table 1:** Endocortical bone histomorphometry. Goldner's trichrome-stained sections were assessed for both static and dynamic endocortical histomorphometry. Osteoid perimeter/bone perimeter, (O.Pm/B.Pm), eroded perimeter/bone perimeter (E.Pm/B.Pm), mineralizing perimeter/bone perimeter (M.Pm/B.Pm), mineralizing perimeter/osteoid perimeter (M.Pm/O.Pm).

Endocortical remodeling parameters	
O.Pm/B.Pm (%)	22.8
E.Pm/B.Pm (%)	67.1
M.Pm/B.Pm (%)	4.7
M.Pm/O.Pm (%)	20.6

### 3.5 The intracortical remodeling appears to be unaffected by the transient denosumab discontinuation

**Fig. 6:**
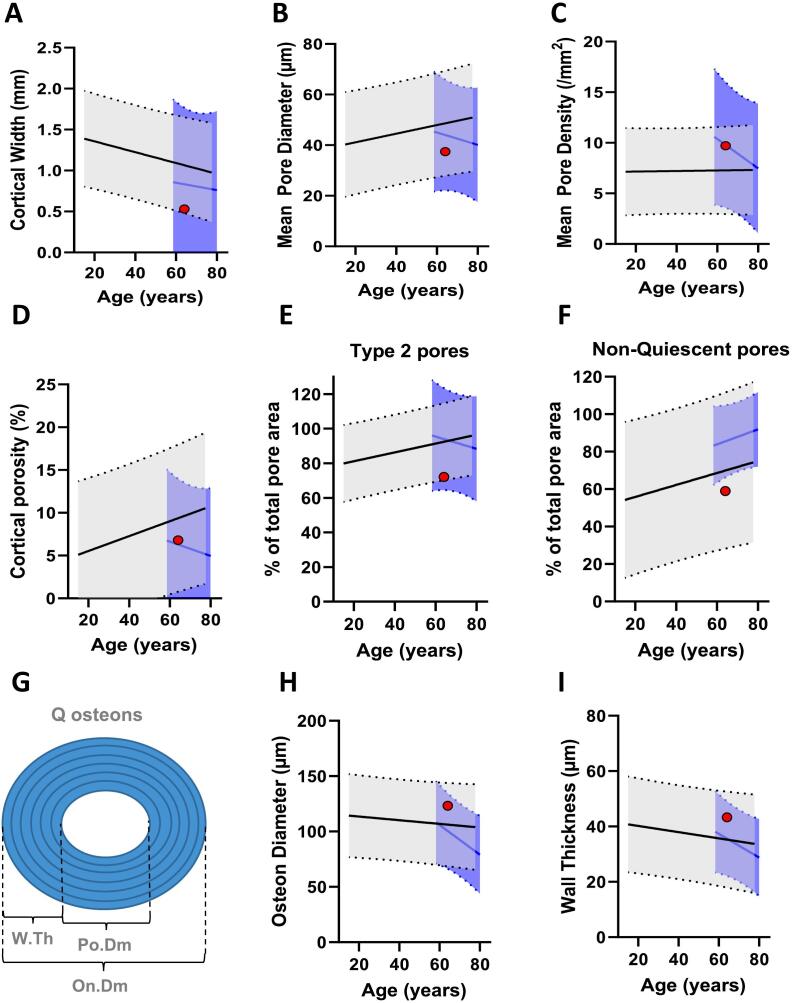
Cortical bone histomorphometry. Goldner's trichrome stained sections were used to assess cortical width (A), mean pore diameter (B), mean pore density (C), cortical porosity (D), type 2 pores' contribution to porosity (E), non-quiescent pores' contribution to porosity (F), osteon diameters (H) and osteonal wall thickness (I), relative to a reference population of female controls and postmenopausal women with osteoporosis, respectively. The black curve represents a reference population of female controls (n = 35) and the blue curve represents a reference population of postmenopausal women with osteoporosis (n = 17), and their dotted lines represent its 95 % prediction bands.

Histomorphometric analysis of the cortical bone compartment revealed a thin cortex width of 0.53 mm, indicating trabecularization and thinning of the cortical compartment (Fig. 6A). Moreover, the analysis revealed a mean pore diameter of 37.4 μm, a mean pore density of 9.7 mm^−2^, and a cortical porosity of 6.8 %. These findings were similar to those of the reference population (Fig. 6B-D). To further assess the remodeling events that may have occurred during the rebound, we sought to investigate pore types and their remodeling stages. This analysis revealed that type 2 pores contributed 72.1 % to the total pore area (Fig. 6E), indicating that the majority of the pores had undergone at least one round of remodeling. Moreover, non-quiescent pores contributed 59 % to the total pore area (Fig. 6F), indicating that intracortical pores had previously been undergoing remodeling. These results did not differ substantially from those of the reference population (Fig. 6E-F). Osteon diameter and wall thickness were 123 μm and 43 μm, respectively – not differing from the reference populations (Fig. 6H-I).

## 4 Discussion

In this clinical case report, effects on bone remodeling and microarchitecture in an iliac bone biopsy were explored in a postmenopausal woman suffering multiple vertebral fractures after denosumab discontinuation. Using histomorphometry and μCT-analysis we found a thin rod-like trabecular bone network along with altered dynamic bone histomorphometry parameters compared to the reference populations.

While several case reports and observational studies have reported effects on systemic bone remodeling markers in serum and bone mineral density after denosumab discontinuation, only a few studies have investigated the local effects of stopping denosumab in bone specimens (Brown et al., 2011; Jahn-Rickert et al., 2020). In a recent clinical, observational study in osteoporosis patients, Jähn-Rickerts and colleagues (Jahn-Rickert et al., 2020) performed histomorphometric analyses of iliac crest biopsies collected from patients that had sustained vertebral fractures after denosumab discontinuation (*n* = 9) compared to treatment naïve patients (*n* = 11) and patients treated with denosumab (*n* = 23). They found significantly a lower trabecular bone volume and near-significant lower trabecular thickness values in the discontinuation group compared to the denosumab-treated group. Our results are generally consistent with these findings since our μCT-analysis revealed a BV/TV value close to the lower 95 % prediction band and trabecular thickness and trabecular separation both below the 95 % prediction bands of the reference population of healthy, non-osteoporotic women. In addition, we found that the trabecular number and the structure model index (a microstructural measure that quantifies the balance between rod-like and plate-like trabeculae) were above the 95 % prediction bands of the reference population. Taken together these results suggest deterioration of the trabecular bone microarchitecture.

Of note, one limitation of the study is the possible presence of a crush artifact indicated by the μCT 3D reconstruction, resulting from the compression of the biopsy. Still, the cellular composition in the bone marrow exhibits no signs of any crushing even though the μCT 3D reconstruction appears crushed. Such an artifact has the potential to influence the assessment of bone micro-architecture, leading to an overestimation in trabecular bone volume, trabecular number, and connectivity density, while underestimating trabecular separation. Despite this limitation, measures such as trabecular thickness, tissue mineral density, and SMI are not expected to be affected by the artifact. Moreover, if the BV/TV value was overestimated due to compression artifacts, the true BV/TV value would have been substantially below the lower 95 % prediction band. We found the connectivity density to be inside the reference range. However, if the biopsy had been crushed the connectivity density of the intact network would have been lower, which is consistent with the qualitative observation of small and disconnected trabeculae.

The histomorphometric analysis revealed both an extended eroded perimeter and an osteoid perimeter, with only a small proportion of the osteoid perimeter undergoing active mineralization. Moreover, the majority of the osteoid bone perimeter was located directly upon eroded-scalloped cement lines with no sign of osteoid mineralization. Similarly, Jähn-Rickerts and colleagues (Jahn-Rickert et al., 2020) found a higher percentage of eroded perimeter and osteoid perimeter in patients discontinuing denosumab treatment compared to treatment naïve and denosumab-treated patients (Jahn-Rickert et al., 2020). In addition, a case study by Maugars et al. that reported multiple vertebral fractures after denosumab discontinuation in a postmenopausal female, also found abnormally extended eroded surfaces at the iliac crest (Maugars et al., 2018). These findings support the proposed pathophysiological mechanism of resorption overshoot after cessation of denosumab. Of note, above mentioned studies reported an exceptionally low ES/BS that was only two-fold higher than the Oc.S/BS (Jahn-Rickert et al., 2020; Maugars et al., 2018), and in part driven by the presence of osteoclasts. Using a strict definition for ES using only the broken lamella structure visualized under polarized light to identify these surfaces, the extent of ES is on average 14–16 % in post-menopausal women (Andreasen et al., 2018a; Andreasen et al., 2018b; Andersen et al., 2013; Andersen et al., 2022). Moreover, it is possible that the extent of the bone resorption following denosumab discontinuation may not only be affected by the duration of denosumab treatment, with longer treatment duration resulting in a more pronounced rebound but may also depend on previous bisphosphonate treatment (Solling et al., 2021).

In the present case, the patient presented with a history of smoking cigarettes and a mild chronic obstructive pulmonary disease treated with inhaled corticosteroids. Although the duration and frequency of smoking are unknown, it has previously been demonstrated that osteoclasts isolated from smokers are less sensitive to zoledronic acid than osteoclasts from nonsmokers (Moller et al., 2020). How smoking affects bone loss following denosumab discontinuation remains unknown, however, cigarette smokers are potentially at higher risk of rebound following discontinuation, despite previous bisphosphonate treatment. Moreover, cigarette smokers may also be less responsive to follow-up bisphosphonate treatment after denosumab discontinuation, rendering this sub-population of patients more challenging to rescue from a rebound.

Surprisingly, our findings are indicative of limited bone formation and delayed mineralization, where remodeling spaces formed during prior resorption were not adequately refilled and mineralized. Moreover, no clear cuboidal bone-forming cells located on the osteoid surfaces were detected on the bone surfaces, indicating that bone formation events had concluded. Whether this limited bone formation and delayed mineralization relate to the transient denosumab discontinuation or its re-initiation remains unclear. Bone resorption is coupled to bone formation to ensure a proper transition from bone resorption to bone formation (Andersen et al., 2013; Lassen et al., 2017). In a study previous study, a single denosumab injection was given to postmenopausal women to identify potential factors that couple bone resorption to bone formation (Weivoda et al., 2020). The study found a significant decrease in genes related to osteoclast differentiation and function, including e.g. *ACP5*, *TNFRSF11A*, and *DCSTAMP*, and genes related to osteoblast differentiation, bone formation, and mineralization, including e.g. *COL1A1*, *BGLAP* and *ALPL (*Weivoda et al., *2020**)*. Thus, the limited bone formation and delayed mineralization observed in the present study could potentially relate to the re-initiation of denosumab treatment. In the case study by Maugars et al., the bone biopsy was obtained 20 months after cessation of denosumab treatment, found normal mineralization indices (Maugars et al., 2018), while in the study by Jähn-Rickerts and colleagues, tetracycline labeling and assessment of mineralization indices were not performed (Jahn-Rickert et al., 2020). There is evidence that alterations in bone remodeling during denosumab treatment and the subsequent discontinuation are reversible since a study in postmenopausal women where iliac crests bone biopsies were obtained 25 months after cessation of denosumab (range 21–29 months, *n* = 15) showed normal bone resorption, formation, and mineralization indices (Brown et al., 2011). However, the duration of denosumab treatment in that study was only 12 months and any potential impacts of long-time suppression of bone turnover per se, which has been observed to impact osteocyte viability and the regulation of bone remodeling, remains uncertain (Jahn-Rickert et al., 2020; Maugars et al., 2018). Finally, it is well known that long-term glucocorticoid treatment and conditions like endogenous Cushing's syndrome may disrupt the balance between bone resorption and bone formation, eventually leading to osteoporosis (Jensen et al., 2015; Jensen et al., 2012). It has previously been demonstrated that glucocorticoid treatment may result in arrested remodeling cycles reflected by an increased occurrence of eroded (reversal) surfaces without any neighboring osteoid surfaces. These arrested reversal surfaces have been characterized by a low cell density and poor transition to bone formation (Jensen et al., 2015). Thus, it cannot be ruled out that the bone loss and reduced osteoblastic activity observed in the present case may also be affected by the inhaled corticosteroids, even though this reflects a very low systemic dose.

In a more recent study by McDonald and colleagues, it has been illustrated in mice that osteoclasts have an alternative cell fate (McDonald et al., 2021). Rather than undergoing apoptosis, mature osteoclasts are capable of recycling, by fissioning into daughter cells, or pre-osteoclasts, called osteomorphs, which upon stimulation are capable of fusing with other pre-osteoclasts, generating mature, bone-resorbing osteoclasts. During RANKL inhibition, recycling is blocked resulting in an accumulation of osteomorphs. As RANKL inhibition diminishes, osteomorphs fuse with neighboring osteomorphs/pre-osteoclasts and become bone-resorbing osteoclasts, eventually leading to an extensive wave of bone resorption (McDonald et al., 2021). A similar mechanism may be responsible for the events occurring following denosumab discontinuation in humans.

While extensive negative effects were observed in the trabecular bone, the intracortical bone compartment appeared relatively unaffected. The reason for these bone compartment-specific effects is unknown but may relate to the proximity of the trabecular bone to the bone marrow from where osteoclasts precursors can be more easily recruited. The sparing of the cortical compartment, as opposed to the trabecular compartment, is in line with clinical observations where several studies report an increased risk of vertebral fractures, while the risk of fractures at skeletal sites predominately comprised of cortical bone – such as the hip – was not elevated (Cummings et al., 2018). However, our histomorphometric analysis revealed an extensively eroded endocortical perimeter and a cortical thickness close to the lower 95 % prediction band, which seems in agreement with previous findings, demonstrating a trend towards a decrease in cortical thickness upon denosumab discontinuation (Jahn-Rickert et al., 2020). Thus, another possible explanation for the seemingly sparing of the intracortical bone could relate to prior trabecularization of the endocortical compartment ultimately leading to cortical bone loss and declining cortical thickness in analogy to changes observed during normal aging (Andreasen et al., 2018a; Bach-Gansmo et al., 2016). Trabecularization of the cortex results from cortical bone resorption with an accumulation of enlarged eroded pores upon existing intracortical pores, which in turn leads to a gradual loss of cortical bone adjacent to the endosteum, where the largest intracortical pores connect into the marrow space, eventually leading to lower cortical thickness (Andreasen et al., 2020; Cooper et al., 2016).

The phenomenon of multiple vertebral fractures with denosumab discontinuation affects only a limited number of those stopping treatment (Tsourdi, 2021). In post hoc analyses of the FREEDOM and FREEDOM extension studies prevalent vertebral fractures, longer time off treatment, greater gain in hip BMD on treatment, and greater loss in hip BMD off treatment were identified as risk factors for multiple vertebral fractures (Cummings et al., 2018; McClung et al., 2017). In addition, observational studies have identified younger age (Solling et al., 2020) and longer treatment duration (Anastasilakis et al., 2017; Sosa-Henríquez et al., 2021) as risk factors for vertebral fractures. The patient in the present case study was relatively young and had been treated with denosumab for five years without any prior observations of vertebral fractures. Although in clinical remission without treatment, the patient had ankylosing spondylitis, which is an auto-inflammatory disease of the spine. This disease may result in an imbalanced RANKL/OPG signaling pathway potentially affecting the differentiation and activation of osteoclasts (Amarasekara et al., 2015). Although speculative, ankylosing spondylitis may have led to more osteoclast recruitment or increased osteoclast function since osteoclast pre-curser cells arise from the macrophage cell lineage. This may have been upregulated as part of a chronic inflammatory condition affecting the spine (Amarasekara et al., 2015), which could have contributed to the clinical presentation. Moreover, osteopenia and osteoporosis are well-known complications of ankylosing spondylitis that may infer an increased risk of vertebral fractures (Klingberg et al., 2012).

Despite the trabecular and endocortical bone surfaces being extensively eroded, no osteoclasts were found present on the bone surface. In addition, the biochemical bone resorption marker CTX was below the median in pre-menopausal women. These findings are likely explained by the fact that an injection with denosumab was given four months before obtaining the bone biopsy, which was chosen as a rescue therapy. Subsequent treatment with either alendronate or zoledronic acid has generally mitigated – but not fully prevented – bone loss after denosumab discontinuation (Solling et al., 2020; Kendler et al., 2020; Makras et al., 2021). Our findings of limited bone formation and delayed mineralization suggest that follow-up treatment with an anti-resorptive pharmaceutical alone may not be sufficient to prevent bone loss after denosumab discontinuation. In postmenopausal osteoporosis, a state that is characterized by high bone turnover – although to a lower degree than after denosumab cessation, the use of combination therapy, with anabolic treatment with Teriparatide and anti-resorptive treatment with Denosumab, has been suggested to be superior to either treatment alone for increasing BMD and improving bone microarchitecture (Tsai et al., 2015; Tsai et al., 2013). Another condition characterized by high bone resorption is disuse osteoporosis. Here, rodent studies have shown that prevention treatment with a combination of PTH (1–34) and zoledronic acid have an additive effect to such a degree that the combination treatment could elevate BV/TV to a level significantly above that of ambulating animals (Vegger et al., 2014). It could further be speculated that treatment with anti-sclerostin antibodies that have a dual anti-resorptive and anabolic effect may be a potentially beneficial treatment option following denosumab discontinuation (Rauner et al., 2021). However, further studies are required to assess the effects of combination therapy with anabolic and anti-resorptive agents for preventing bone loss after denosumab discontinuation.

In conclusion, we present a case report of multiple vertebral fractures after denosumab discontinuation with histomorphometric evidence that denosumab discontinuation leads to extensive bone resorption, preferentially of the trabecular bone compartment leading not only to a deteriorated and structurally altered trabecular network but also to signs of a limited trabecular bone formation and delayed mineralization. This highlights the importance of developing the necessary tools to identify patients at risk of rebound upon denosumab discontinuation and developing an optimal discontinuation strategy for patients that are to discontinue treatment. Thus, the optimal use of anti-osteoporosis therapies in this setting requires further investigation.

## Funding

Laboratory costs were covered by 10.13039/100008397The Velux Foundation (Grant no. 25723). The μCT scanner was donated by the 10.13039/100007214Velux Foundation. The salary for Ph.D. student Bilal El-Masri was covered by 10.13039/501100004196Odense University Hospital's Ph.D. fund (Grant no. 4038), The Region of Southern Denmark's Ph.D. fund (Grant no. 731), and The Novo Nordic Foundation (Grant no. 0069586). The salary for Ph.D. Student Lisbeth Koch Thomsen was covered by the Dept. of Clinical Research, 10.13039/501100006356University of Southern Denmark, and The Region of Southern Denmark's Ph.D. fund (Grant no. 373).

## Code availability

None.

## CRediT authorship contribution statement

Study design: LDJ, SH, TLA. Data collection: LDJ, JST, CE, BME, TLA. Data interpretation: LDJ, BME, CMA, LKT, JST, CE, TLA, SH. Drafting manuscript: LDJ, BME, SH, TLA. Revising manuscript: All authors. Approving final version of manuscript: All authors.

## Declaration of competing interest

TLA and CMA have collaborated with Amgen on the cortical effect of denosumab in the FREEDOM study.

## Data Availability

Data will be made available on request.
